# Spatial and Temporal Variations of Polar Ionospheric Total Electron Content over Nearly Thirteen Years

**DOI:** 10.3390/s20020540

**Published:** 2020-01-19

**Authors:** Hui Xi, Hu Jiang, Jiachun An, Zemin Wang, Xueyong Xu, Houxuan Yan, Can Feng

**Affiliations:** 1 Institute of Earth Sciences, Academia Sinica, Taipei 11529, Taiwan; xihui@earth.sinica.edu.tw; 2 North Information Control Research Academy Group Co., Ltd., Nanjing 211153, China; xxyyeah@163.com (X.X.); yhx412@126.com (H.Y.); fengcan2004@126.com (C.F.); 3 Chinese Antarctic Center of Surveying and Mapping, Wuhan University, Wuhan 430079, China; jcan@whu.edu.cn (J.A.); zmwang@whu.edu.cn (Z.W.)

**Keywords:** global navigation satellite system (GNSS), total electron content (TEC), polar ionosphere, spherical cap harmonic (SCH) model, ionization patch

## Abstract

It is of great significance for the global navigation satellite system (GNSS) service to detect the polar ionospheric total electron content (TEC) and its variations, particularly under disturbed ionosphere conditions, including different phases of solar activity, the polar day and night alternation, the Weddell Sea anomaly (WSA) as well as geomagnetic storms. In this paper, four different models are utilized to map the ionospheric TEC over the Arctic and Antarctic for about one solar cycle: the polynomial (POLY) model, the generalized trigonometric series function (GTSF) model, the spherical harmonic (SH) model, and the spherical cap harmonic (SCH) model. Compared to other models, the SCH model has the best performance with ±0.8 TECU of residual mean value and 1.5–3.5 TECU of root mean square error. The spatiotemporal distributions and variations of the polar ionospheric TEC are investigated and compared under different ionosphere conditions in the Arctic and Antarctic. The results show that the solar activity significantly affects the TEC variations. During polar days, the ionospheric TEC is more active than it is during polar nights. In polar days over the Antarctic, the maximum value of TEC always appears at night in the Antarctic Peninsula and Weddell Sea area affected by the WSA. In the same year, the ionospheric TEC of the Antarctic has a larger amplitude of annual variation than that of the TEC in the Arctic. In addition, the evolution of the ionization patch during a geomagnetic storm over the Antarctic can be clearly tracked employing the SCH model, which appears to be adequate for mapping the polar TEC, and provides a sound basis for further automatic identification of ionization patches.

## 1. Introduction

The polar regions namely the Arctic and the Antarctic are the locations of the geographical and geomagnetic poles. The magnetic field lines over the polar caps penetrate to the Earth or extend outward to connect with the interplanetary magnetic field. The large-scale magnetospheric electric field, formed by the interaction between the magnetosphere and solar wind, is mapped to the high-latitude ionosphere along the magnetic field lines with high conductivity; meanwhile, changes in the ionosphere can also modulate the magnetospheric electric field. These interactions result in a highly variable polar ionosphere. The total electron content (TEC) is one of the significant parameters of the ionosphere, which represents the integration of the electron density along the path of the radio signal [1,2,3]. The ionospheric delay error of the radio signal is directly proportional to the TEC [3]. Therefore, it is not only of great scientific significance to detect the spatiotemporal distribution and variation of the polar ionospheric TEC, but also of great guiding significance to the global navigation satellite system (GNSS) high-precision positioning, navigation, timing (PNT) service, radio communication, and other human space activities in the polar regions.

The study of the polar ionospheric variation requires accurate ionospheric models. The most commonly used ones include broadcast ionospheric models, empirical ionospheric models, and global ionospheric map (GIM) models provided by the International GNSS Service (IGS) [4,5,6,7]. However, they have degraded accuracy in mapping the ionospheric TEC of the polar regions [7]. Thanks to rapid development, GNSS, measurements can now provide adequate accuracy for mapping the polar TEC and have become the widely used method for ionospheric research [2,8,9,10]. According to the dispersive properties of the ionosphere, the TEC along a given path from the receiver to the satellite can be estimated by using dual-frequency or multifrequency GNSS measurements, and the regional TEC can be further mapped with different ionospheric models. The commonly used models for regional TEC mapping mainly include the polynomial (POLY) function model, the generalized trigonometric series function (GTSF) model, the spherical harmonic (SH) function model, and the spherical cap harmonic (SCH) function model [3,11,12,13,14,15]. These models have their own advantages and disadvantages, and they have optimal performances only under the appropriate conditions. Liu et al. [12] evaluated the applicability of these ionospheric models in the Arctic region. An et al. [16] mapped the Antarctic ionospheric TEC and analyzed the variation characteristics using the SCH model.

Due to the special geographical location of the polar regions, here the ionosphere is affected by variable phenomena, such as the polar day and night alternation, the Weddell Sea anomaly (WSA), and ionospheric storms. Polar day indicates the phenomenon in which the Sun stays above the horizon for more than 24 h, while polar night refers to night lasting for more than 24 h. The WSA means that the electronic density at nighttime is larger than that in the daytime over the regions of Antarctic Peninsula and Weddell Sea in summer. The maximum of the daily electronic density occurs from 22:00 to 4:00 local time (LT) and the minimum appears from 12:00 to 18:00 LT. The polar days and nights as well as the WSA have significant impacts on ionospheric models [7]. When an ionospheric storm occurs, ionization patches are often present. They are formed in the F layer, and their electronic density is more than twice than that of the surrounding areas [17,18]. The ionization patches lead to several difficulties with regard to radio signals, including phase advance, time delay, and Doppler shifts [19]. The movements of the ionization patches and associated density gradients have severe interference on high-frequency radio communication and satellite navigation. In addition, the edges of the ionization patches may generate some irregularities, resulting in serious ionospheric scintillation that may interrupt satellite communication and navigation systems [20,21].

The aforementioned phenomena have been vastly observed [7,12,21,22,23,24]. Bellchambers and Piggott [25] first found the WSA in 1957 using the ionosonde measurements. The WSA may originate in the South Pacific region and has an important relationship with the ionospheric equatorial anomalies [20,23]. With the developments of satellite technologies, the GNSS measurements [12], altimetry satellite [26], and occultation observation [27] provide abundant data to study the WSA and its impact on polar ionosphere. Jiang et al. [7] tested the performances of different ionospheric models under WSA conditions. In addition, it is of great significance to study the formation and evolution of ionization patches [28]. Coley and Heelis [22] compared the differences in the ionization patches’ variations with seasons, time, and interplanetary magnetic field in the Arctic and Antarctic. Crowley et al. [29] detected their evolution and dissipation in the aurora zone at night based on multisource data. Zhang et al. [30] utilized the Super Dual Auroral Radar Network (SuperDARN) to track the generation and evolution of such patches in the Arctic during a magnetic storm on September 24 in 2011, while Liu et al. [12] verified their evolution on that particular day by using global positioning system (GPS) ground observations.

At present, the research on the polar ionosphere is mostly based on ground radar and occultation observation. Although these observations can obtain detailed ionospheric information, they are expensive, weakly time effective, and cannot cover the entire polar regions. GNSS measurements have also been applied to the research of polar ionosphere, but most of the existing studies are based on short-term observations, and there are no long-term analyses. Moreover, the polar regions are highly variable environments, which are affected by solar activity, the polar days and nights, the WSA, and the presence of geomagnetic storms. Few studies have investigated the variations of the polar ionospheric TEC under such complicated influences. Lastly, most of the previous studies only focus on one polar region, either Arctic or Antarctic, and few studies analyze and compare the variation of the polar TEC in both these regions and the coupling relationship between them.

In this paper, we mainly focus on the spatiotemporal distributions and variations of the polar ionospheric TEC under disturbed conditions, including different periods of solar activity, the polar day/night alternation, the WSA as well as the presence of geomagnetic storms, in both the Arctic and the Antarctic for about 13 years. As a first step, the GNSS measurements are adopted to map the polar ionospheric TEC for about one solar cycle using four models (the POLY model, the GTSF model, the SH model, and the SCH model), comparing their performances in mapping the polar TEC. Next, the spatial characteristics of the polar ionospheric TEC are analyzed under comprehensive disturbed conditions. Then, the temporal variations of the polar TEC and their correlations with GIM and solar activity index of F10.7 are investigated. Finally, we track the generation and evolution of the ionization patches during a magnetic storm in the Antarctic on day of year (DOY) 076 in 2015.

## 2. Materials and Methods

### 2.1. Data Source

**Figure 1:**
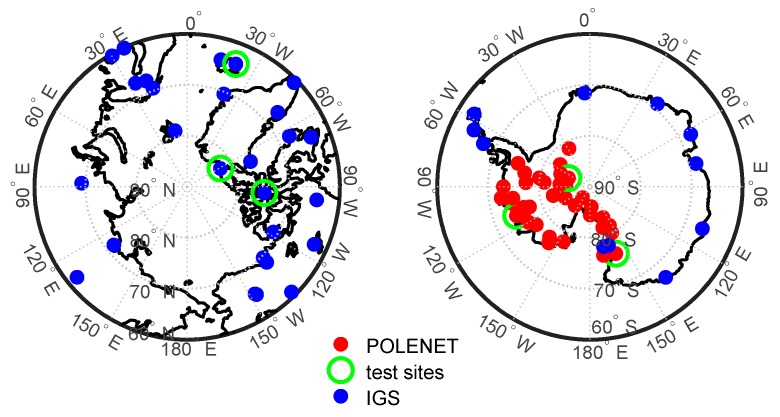
The geographical locations of global positioning system (GPS) tracking stations, including the International GNSS (global navigation satellite system) Service (IGS) stations (blue dots), the Polar Earth Observing Network (POLENET) stations (red dots), and the verification stations (green circles) in the Arctic (**left**) and Antarctic (**right**).

GPS data provided by the IGS and the POLENET (Polar Earth Observing Network) were used in this study. Figure 1 shows the geographical locations of GPS tracking stations in the polar regions, including the IGS stations (blue dots), the POLENET stations (red dots), and the verification stations (green circles) which are used for verifying the ionospheric TEC models. In the Arctic, there are 46 IGS stations available, while there are 10 IGS stations and 44 POLENET stations in the Antarctic. Most POLENET stations have provided measurements since 2010. Before 2010, only a few stations with non-uniform distribution were available in the Antarctic, which were insufficient to map the distributions and variations of the TEC; as a consequence, the modeling of the ionospheric TEC in the Antarctic region started only from 2010. The sampling rate of the GPS measurements was 30 s. The satellite positions were calculated according to the broadcast ephemeris file (brdc) provided by the Crustal Dynamics Data Information System (CDDIS).

### 2.2. Deriving TEC from GPS Measurements

The ionospheric model is established in the polar regions, which requires high-precision ionosphere TEC observations. The said observations obtained from the geometry-free combination of dual-frequency pseudo-range measurement suffer from noise and multipath effects [31]. In terms of the carrier phase measurements, it is difficult to quantify the unknown integer phase ambiguities. Therefore, a carrier-to-code leveling (CCL) approach was used to calculate the ionosphere TEC observations [14], which, derived from CCL method, have the expression shown in Equation (1).

(1) $${\hat{I}}_{r}^{s}={\mathrm{STEC}}_{r}^{s}-\vartheta ({\mathrm{DCB}}_{r}+{\mathrm{DCB}}^{s})+\varepsilon_{\hat{I}},$$

where ${\hat{I}}_{r}^{s}$ is the ionospheric observation in TEC unit (TECU); s and r denote the pseudo-random noise (PRN) of the given satellite and receiver, respectively; $\vartheta =\frac{c}{\alpha (f_{i}^{-2}-f_{j}^{-2})}$ is a constant used to convert the time unit (second) to TECU, where $\alpha =4.028\times 10^{17}\;{\mathrm{ms}}^{-2}{\mathrm{TECU}}^{-1}$, c is the speed of light, f is the frequency; ${\mathrm{DCB}}_{r}$ and ${\mathrm{DCB}}^{s}$ are receiver and satellite differential code biases (DCBs).

**Figure 2:**
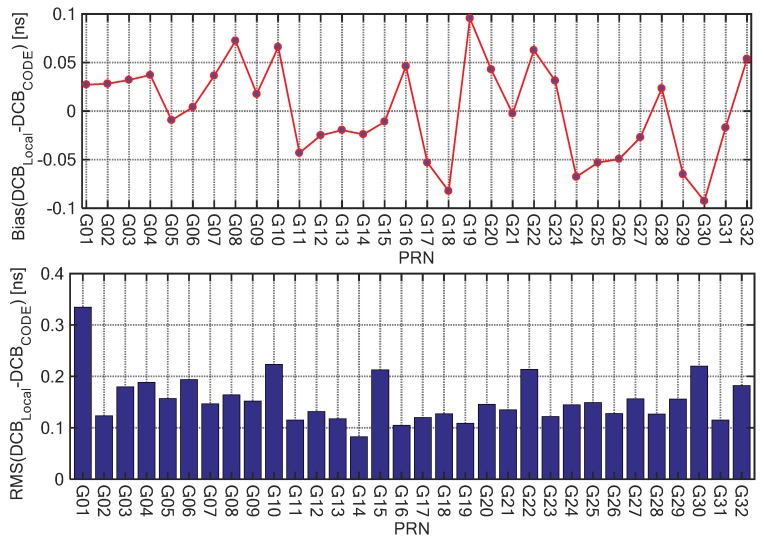
Bias (**top**) and root mean square (RMS) errors (**bottom**) of the differences between the satellite differential code biases (DCBs) estimated by the IGGDCB method (Institute of Geodesy and Geophysics) and the monthly P1-P2 DCBs products provided by the Center for Orbit Determination in Europe (CODE) at the individual GPS satellites as a function of the pseudo-random noise (PRN).

The TEC derived from GPS measurements contains the satellite and receiver DCBs [32]. In this paper, the satellite DCBs of P1-C1 were provided by the Center for Orbit Determination in Europe (CODE). Since P1-P2 DCB products provided by IGS only have receiver DCBs of IGS tracking stations, the GPS satellite and receiver DCBs of P1-P2 were computed employing the IGGDCB (IGG, Institute of Geodesy and Geophysics, Wuhan, China) method based on all GPS stations shown in Figure 1. Figure 2 shows the bias, upper panel, and root mean square (RMS) errors, bottom panel, of the differences between the satellite DCBs estimated by the IGGDCB method and the monthly P1-P2 DCBs products provided by the CODE. Compared with the DCB products of CODE, the bias of IGGDCB-based satellite P1-P2 DCB estimates was determined at the level of ±0.1; the maximum RMS error was 0.34 ns, while the average appeared to be less than 0.2 ns. The DCBs of satellite and receiver were taken into Equation (1) to obtain the pure TEC value.

For this study, in order to mitigate the influence of spatial gradients on ionospheric mapping [33] and multipath effect, we only adopted the slant TEC data corresponding to the line-of-sight (LOS) with a cutoff angle of 20° at the observation point. Instead of modeling the ionosphere as being distributed in altitude, the thin shell model was used for mapping the polar ionospheric TEC at the height of 425 km.

## 3. Mapping Performances of the Four Models over the Arctic and Antarctic

According to the existing research, the commonly used ionospheric TEC models have degraded accuracy in the polar regions [7]. Thus, in order to more reliably analyze its characteristics, the GPS ground observations were adopted for the mapping procedure.

### 3.1. Establishment of Four Ionospheric TEC Models

In this section, four models were considered to map the polar ionospheric TEC, including the POLY model, the GTSF model, the SH model, and the SCH model. A brief introduction of these four models is given in the following paragraphs.

The POLY model has a simple structure and is mainly used for regional TEC mapping. In this model, the TEC value is regarded as a series function of various factors, such as sun angle and latitude [14]. Its expression is as follows:

(2) $$\mathrm{VTEC}(\varphi ,\lambda ,t)=\sum_{i=0}^{n}\sum_{j=0}^{m}A_{ik}{\left(\varphi -\varphi_{0}\right)}^{i}{\left(\lambda -\lambda_{0}+t-t_{0}\right)}^{j},$$

where VTEC(φ,λ,t) is the vertical TEC (VTEC) at the ionosphere pierce point (IPP) (φ,λ) at time t; n and m are the degree and order of the model, respectively; $A_{ik}$ are the unknown model coefficients; $\left(\varphi_{0},\lambda_{0}\right)$ are the geographic latitude and longitude at the regional center; $t_{0}$ is the sun angle corresponding to the middle time of the mapping period.

According to the spatiotemporal characteristics of regional ionospheric TEC, Yuan and Ou [11] used both the polynomial function related to latitude and LT and the trigonometric series function related to LT to map the diurnal variation of VTEC, which improved the simulation capabilities of regional TEC. This model is developed on the basis of a trigonometric series function model and is called GTSF model. The expression is as follows:

(3) $$\{\begin{array}{c} \mathrm{VTEC}(\varphi ,\lambda ,t)=\sum_{i=0}^{N_{I}}\sum_{j=0}^{N_{J}}A_{i}{}_{j}\varphi_{m}{}^{i}h^{j}+\sum_{k=0}^{N_{K}}\left(B_{k}\cos(kh)+C_{k}\cos(kh)\right)\\ \varphi_{m}=\varphi +0.064\cos(\lambda -1.617)\\ h=2\pi (t-14)/T,\;T=24\;h \end{array},$$

where $N_{I}$ and $N_{J}$ are the maximum degree and order of the polynomial function, respectively; $N_{K}$ is the maximum degree of the trigonometric series function; $A_{i}{}_{j}$, $B_{k}$, $C_{k}$ are the model coefficients to be estimated; $\varphi_{m}$ is the geomagnetic latitude at IPP; h is the variable related to LT.

The SH model can precisely map the global ionospheric TEC [3]. In regional mapping, the basis functions of SH do not have orthogonality. However, the lower-order SH model still can be used for regional TEC mapping with high accuracy [16]. The function model is as follows:

(4) $$VTEC(\varphi ,\lambda )=\sum_{n=0}^{n_{\max}}\sum_{m=0}^{m_{\max}}P_{nm}(\sin\varphi )\cdot (A_{nm}\cos(m\lambda )+B_{nm}\sin(m\lambda )),$$

where VTEC(φ,λ) is the VTEC at the geomagnetic coordinate (φ,λ) of the IPP; $n_{\max}$ and $m_{\max}$ are the maximum degree and order of the SH function, respectively; P(sinφ) is the Legendre function; $A_{nm}$ and $B_{nm}$ are the model coefficients to be estimated.

Because the basis functions of the SH model are not orthogonal in regional mapping, the SCH functions were introduced. The SCH model consists of a set of functions obtained by solving a Laplace’s equation on a specific spherical cap [12]. For regional modeling, the zero-order term indicates the average TEC within the local area. The function expression of the SCH model is similar to that of the SH model; the main difference between them is that the SCH model replaces the integer-order Legendre function with a non-integer Legendre function. The expression is the following:

(5) $$VTEC(\varphi_{c},\lambda_{c})=\sum_{n=0}^{n_{\max}}\sum_{m=0}^{m_{\max}}{\tilde{P}}_{nm}(\cos\varphi_{c})\cdot ({\tilde{C}}_{nm}\cos(m\lambda_{c})+{\tilde{S}}_{nm}\sin(m\lambda_{c})),$$

where $\mathrm{VTEC}(\varphi_{c},\lambda_{c})$ represents the VTEC at the spherical cap coordinate $\left(\varphi_{c},\lambda_{c}\right)$ of the IPP; $n_{\max}$ and $m_{\max}$ are the maximum degree and order of the series, respectively; $\tilde{P}(\cos\varphi )$ is the normalized associated Legendre function; ${\tilde{C}}_{nm}$ and ${\tilde{S}}_{nm}$ are the SCH coefficients to be estimated.

Since the ionospheric TEC is directly connected to the LT, 24 h of one calendar day was evenly divided into 12 sessions. Each session was fitted using four ionospheric models, respectively. According to the characteristic of each model, different parameter configurations were used in the study. For the POLY model, the mapping was based on the Earth-fixed geographical coordinate system with degree of 5 and order of 6. Thus, the total number of model parameters was 30 in each session. The GTSF model can map the diurnal TEC variation through a set of parameters [11]. We adopted a 19-parameter GTSF model to map the polar diurnal TEC. The model was based on the Earth-fixed geomagnetic coordinate system. For the SH model, the mapping was based on the solar geomagnetic coordinate system. The degree and order were 5, and the number of model parameters was 36 in each session. For the SCH model, the simulation was based on the Earth-fixed geomagnetic coordinate system. The maximum degree and order were 6, and there were 49 model parameters to be estimated in each session. The half angle of the spherical cap was 25°.

### 3.2. Evaluation of the Four Ionosphere TEC Models

**Figure 3:**
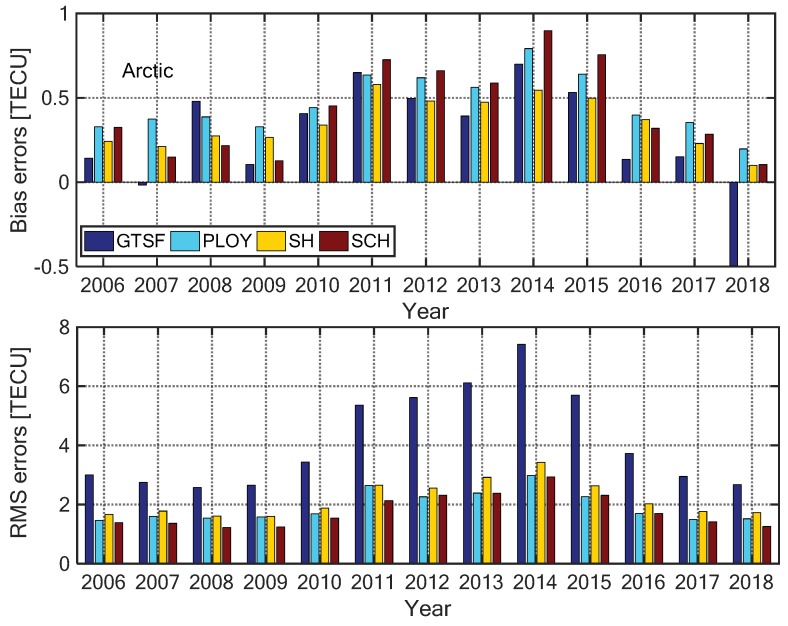
Annually ionospheric total electron content (TEC) mapping bias (**top**) and RMS (**bottom**) errors of the four models in the Arctic from 2006 to 2018.

**Figure 4:**
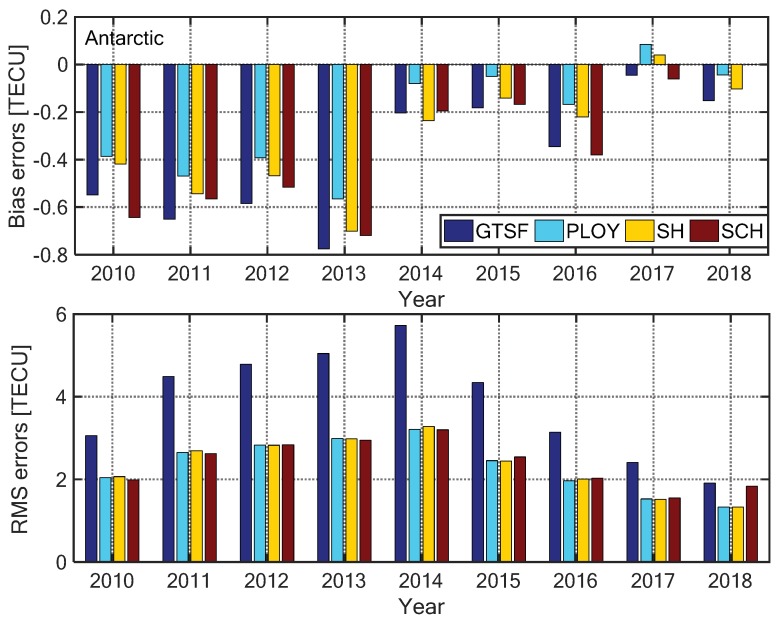
Annually ionospheric TEC mapping bias (**top**) and RMS (**bottom**) errors of the four models in the Antarctic from 2010 to 2018.

In this section, we compare the performances of the four models for mapping the polar ionospheric TEC. Figure 3 and Figure 4 present the annual mapping bias errors and RMS errors of the four models for the Arctic and Antarctic regions, respectively. The statistical results were based on the GPS-TEC provided by the verification stations shown in Figure 1. It can be seen from the figures that the bias errors of the four models are less than 0.8 TECU (1 TECU = 10^16^ electrons m^−2^). In the Arctic, the model bias errors were basically positive, while most of the biases were negative in the Antarctic, indicating that the models overestimate the true TEC in the Arctic and underestimate it in the Antarctic. Moreover, the GTSF model had the largest RMS errors, while the other three models had mapping RMS errors between 1.5 and 3.8 TECU. The SCH model presented the best performance, as already found by Liu et al. [12] in the Arctic. Here the RMS errors of the SCH model were less than 3.0 TECU, while in the Antarctic, the RMS errors were within 1.5–3.5 TECU and the model itself had lower mapping accuracy with respect to the Arctic; this was because of the non-uniform distribution of GPS observation stations shown in Figure 1.

## 4. Spatial and Temporal Variation Characteristics of TEC in the Polar Regions

The previous section has shown that the SCH model has higher mapping accuracy with respect to the three models for the polar regions during about one solar cycle. Therefore, starting from this section, we analyze the spatial and temporal variations of the polar ionospheric TEC based on the SCH model solely.

### 4.1. Spatial Distribution Characteristics of Ionosphere TEC in the Polar Regions

In order to analyze the spatiotemporal characteristics of the polar TEC under disturbed conditions, including different phases of solar activity, the polar days and nights, and the WSA, days of year (DOYs) 001 and 185 (corresponding to January 1 and July 4) in 2010 (low solar activity year) and 2014 (high solar activity year) were selected in this paper. The solar activity index of F10.7 on DOYs 001 and 185 in 2010 were 75.2 and 71.6 sfu, respectively, and in 2014 were 159.6 and 116.8 sfu, respectively. DOYs 001 and 185 were polar night and polar day in the Arctic, while in the Antarctic they were polar day and polar night, respectively. Moreover, the polar day on DOY 001 in the Antarctic was accompanied by the WSA.

**Figure 5:**
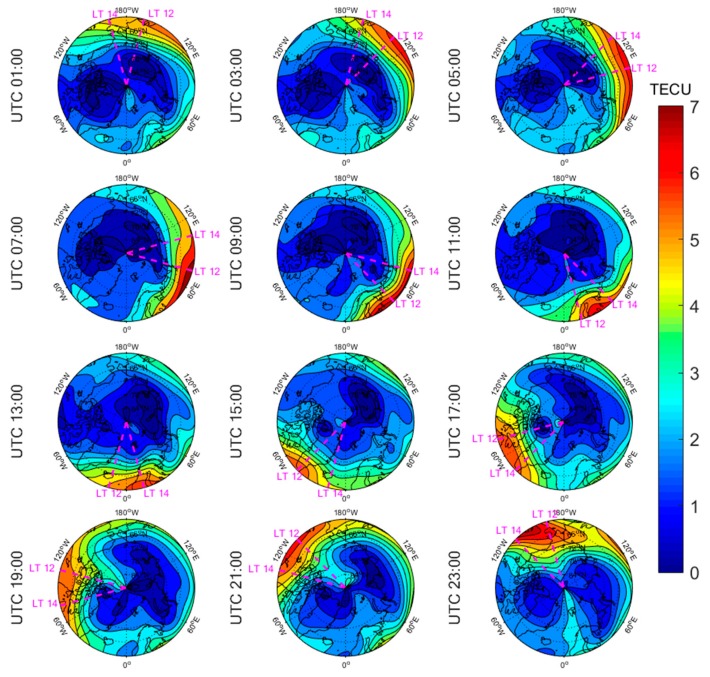
Spatial distribution of mapping TEC of 12 sections under low solar activity year, polar night (DOY 001 in 2010) over the Arctic. The magenta lines in each subplot represent the longitudes at 12:00 and 14:00 at the middle of the 2 h section of the corresponding subplot.

**Figure 6:**
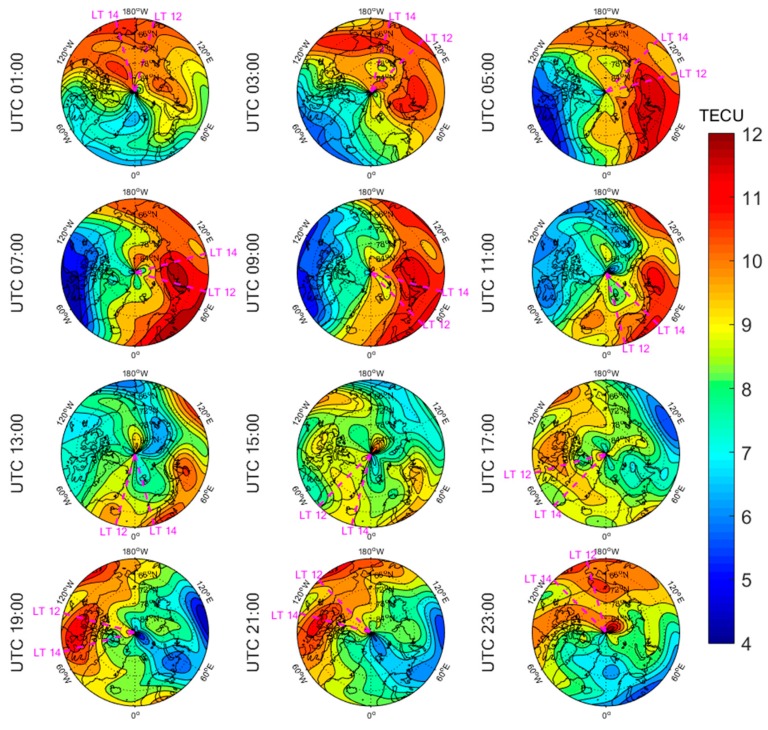
Spatial distribution of mapping TEC of 12 sections under low solar activity year, polar day (DOY 185 in 2010) over the Arctic. The magenta lines in each subplot represent the longitudes at 12:00 and 14:00 at the middle of the 2 h section of the corresponding subplot.

**Figure 7:**
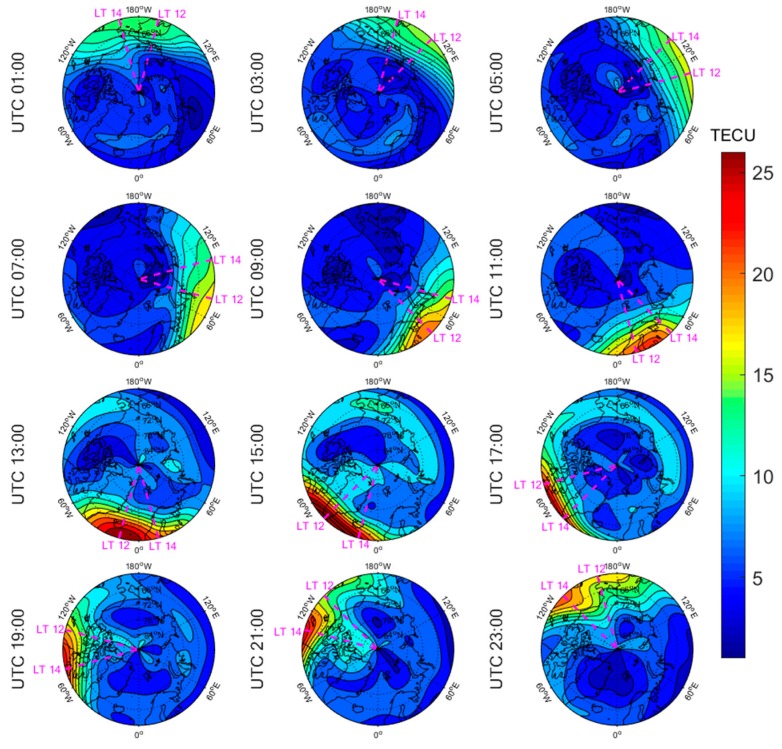
Spatial distribution of mapping TEC of 12 sections under high solar activity year, polar night (DOY 001 in 2014) over the Arctic. The magenta lines in each subplot represent the longitudes at 12:00 and 14:00 at the middle of the 2 h section of the corresponding subplot.

**Figure 8:**
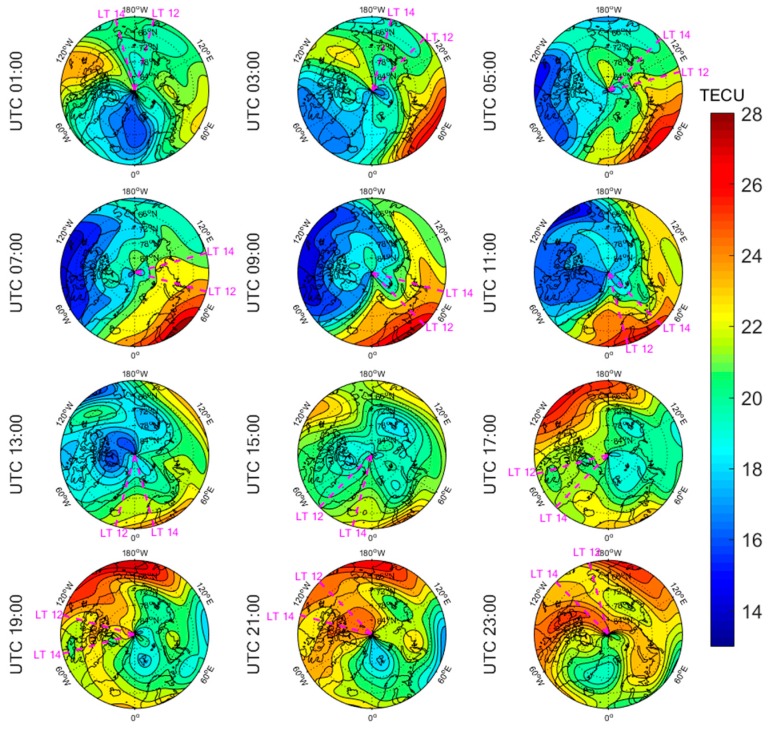
Spatial distribution of mapping TEC of 12 sections under high solar activity year, polar day (DOY 185 in 2014) over the Arctic. The magenta lines in each subplot represent the longitudes at 12:00 and 14:00 at the middle of the 2 h section of the corresponding subplot.

**Figure 9:**
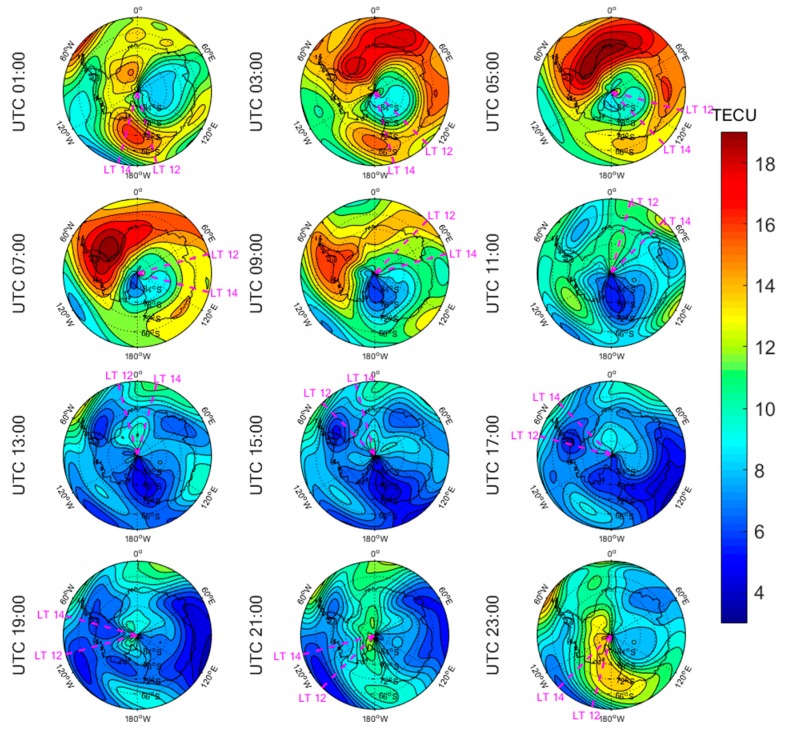
Spatial distribution of mapping TEC of 12 sections under low solar activity year, polar day (DOY 001 in 2010) over the Antarctic. The magenta lines in each subplot represent the longitudes at 12:00 and 14:00 at the middle of the 2 h section of the corresponding subplot.

**Figure 10:**
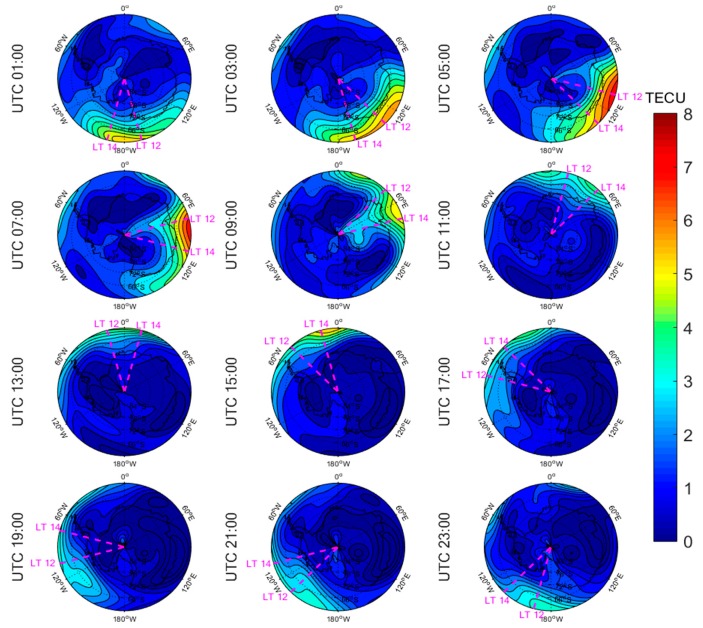
Spatial distribution of mapping TEC of 12 sections under low solar activity year, polar night (DOY 185 in 2010) over the Antarctic. The magenta lines in each subplot represent the longitudes at 12:00 and 14:00 at the middle of the 2 h section of the corresponding subplot.

**Figure 11:**
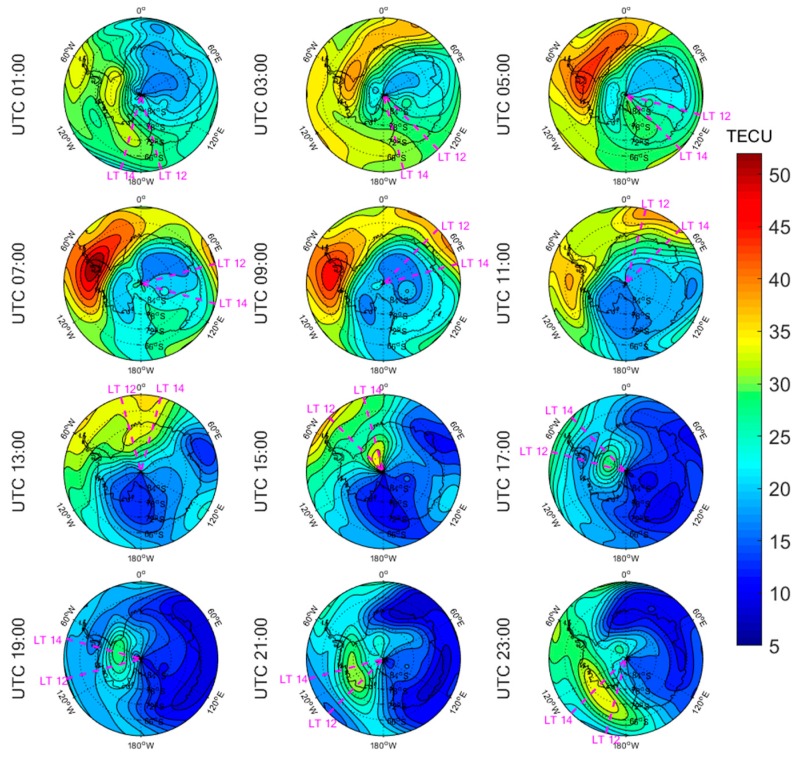
Spatial distribution of mapping TEC of 12 sections under high solar activity year, polar day (DOY 001 in 2014) over the Antarctic. The magenta lines in each subplot represent the longitudes at 12:00 and 14:00 at the middle of the 2 h section of the corresponding subplot.

**Figure 12:**
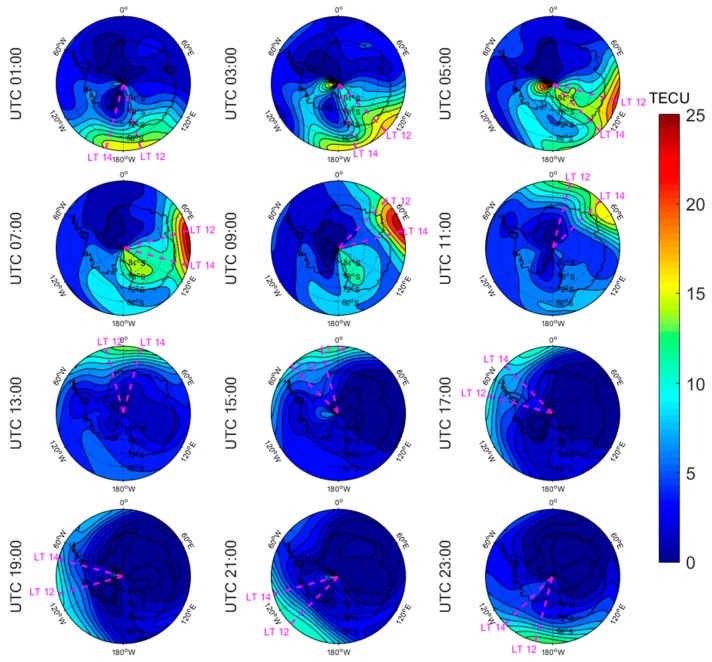
Spatial distribution of mapping TEC of 12 sections under high solar activity year, polar night (DOY 185 in 2014) over the Antarctic. The magenta lines in each subplot represent the longitudes at 12:00 and 14:00 at the middle of the 2 h section of the corresponding subplot.

Figure 5, Figure 6, Figure 7, Figure 8, Figure 9, Figure 10, Figure 11 and Figure 12 show the spatial distributions of the ionospheric TEC on DOYs 001 and 185 in 2010 and 2014 over the Arctic and Antarctic. As shown in the figures, the solar activity significantly affected the changes of the ionospheric TEC. For the same DOY and region, the overall ionospheric TEC level during the high solar activity year was generally three times higher than that during the low solar activity year (Figure 5 vs. Figure 7; Figure 6 vs. Figure 8; Figure 9 vs. Figure 11; Figure 10 vs. Figure 12). In addition, the ionosphere TEC had different characteristics during polar days and nights. In the same year and region, the ionospheric TEC underwent a more active condition in polar days than in polar nights. For example, the solar activity levels were similar on DOYs 001 and 185 in 2010, but the TEC in the polar day was about twice as high as that in polar night in the same region. The main reason is that during days, there is more solar radiation within one day in most of the areas, and the amount of neutral gas ionization in the ionosphere increases. On the contrary, during polar nights, most of the region has no solar radiation in a single day at all, the neutral gas cannot be ionized and the free electrons are constantly depleted. The main source of ionization in the polar nights is from the high-energy particles which impinge on the ionosphere due to the extended magnetic field lines in the magnetosphere [34].

The TEC values at different latitudes varied widely at the same time and generally increased with the decrease of latitude. However, during Arctic polar days, the TEC decreased with decreasing latitude at night (Figure 6 and Figure 8). This was due to the fact that there was more solar radiation in high-latitude areas within a day during polar days. At night, the solar radiation weakened as the latitude decreased during polar days, and the generation of free electrons diminished. Similarly, during polar nights, the ionospheric TEC significantly increased over the Arctic and Antarctic when the latitude became lower in the daytime (Figure 5, Figure 7, Figure 10 and Figure 12).

The polar ionospheric TEC exhibits significant diurnal variations, which are clearly shown during polar days (Figure 6 and Figure 8) and polar nights in the Arctic (Figure 5 and Figure 7), as well as during polar nights in the Antarctic (Figure 10 and Figure 12). At a given time, the ionospheric TEC value generally reached its maximum from 12:00 to 14:00 LT. However, in the Antarctic, during polar days (Figure 9 and Figure 11), the TEC at UTC (Universal Time Coordinated) from 1:00 to 11:00 did not reach the maximum value near the time window 12:00–14:00 LT. During this period, the maximum value of TEC mostly appeared in the areas of the Antarctic Peninsula and the Weddell Sea. This was mainly caused by the WSA, which is characterized by a greater electronic density at nighttime with respect to daytime in summer over the Antarctic Peninsula and Weddell Sea areas [23]. As a consequence, during the polar days in the Antarctic, the most active ionospheric TEC occurred at night in the Antarctic Peninsula and the Weddell Sea.

### 4.2. Temporal Variation Characteristics of Ionosphere TEC in the Polar Regions

**Figure 13:**
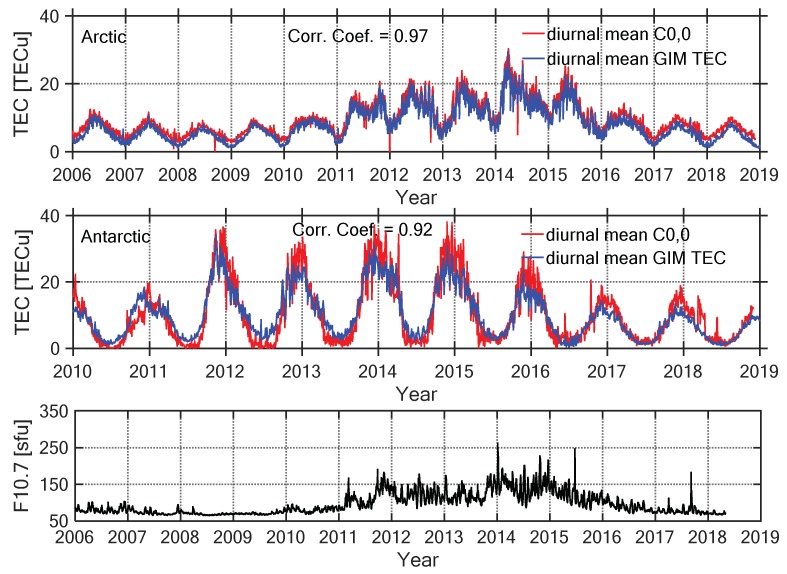
Mean diurnal variations of the C0,0 and the global ionospheric map (GIM) TEC in the Arctic (**top**), Antarctic (**middle**), and the time series of the solar activity index of F10.7 (**bottom**).

The C0,0 coefficient (zero-order term of the SCH coefficients) of the SCH model in each session represents the average regional TEC value [12]. Figure 13 illustrates the mean diurnal variations of the C0,0, the CODE GIM models, and the solar activity index of F10.7. Because of an insufficient number of GNSS tracking stations with uneven distribution in the Antarctic before 2010, the ionospheric TEC cannot be effectively modeled. Thus, the available TEC model in the Antarctic starts from 2010. As seen in Figure 13, the mean diurnal variation of C0,0 coefficient was very close to that of the GIM model. The correlation coefficients between them were 0.97 in the Arctic, and 0.92 in the Antarctic, respectively. During about one solar cycle, the ionospheric TEC variation was strongly correlated with the solar activity level. From 2006 to 2018, the solar activity reached its highest level in 2014, and the maximum values of the ionospheric TEC also occurred in that particular year over the Antarctic and Arctic. The ionospheric TEC of the Antarctic had a larger amplitude of annual variation than that of the TEC in the Arctic. Moreover, the TEC variation of the Arctic during one year was opposite to that of the Antarctic. In the Arctic, the ionospheric TEC reached the maximum in June and the minimum in December; on the contrary, the maximum appeared in December and the minimum occurred in June over the Antarctic. This was directly connected to the seasonal variation of the ionosphere.

**Figure 14:**
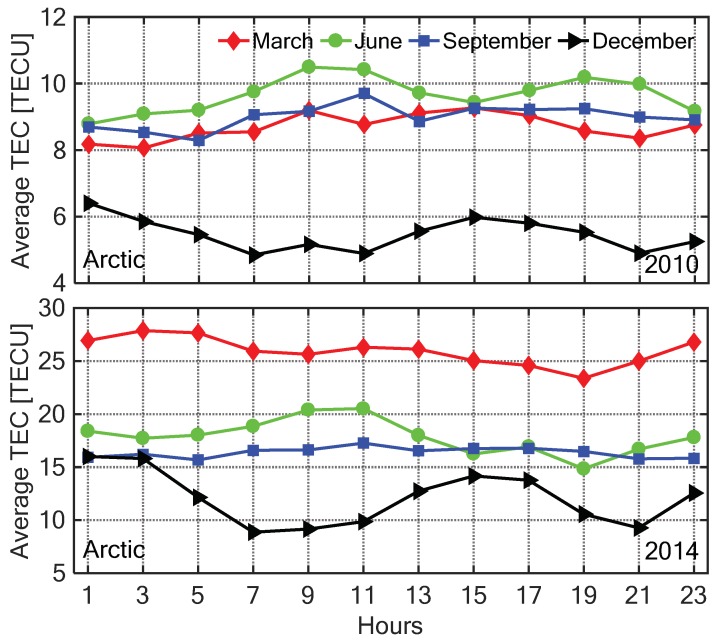
The time series of the C0,0 coefficient with 2 h resolution in March, June, September, and December under low (**top**) and high (**bottom**) solar activity year in the Arctic.

**Figure 15:**
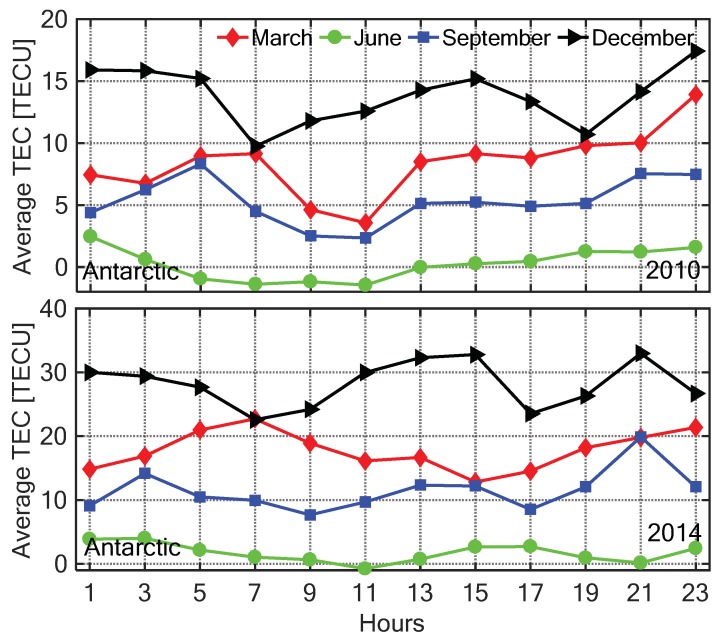
The time series of the C0,0 coefficient with 2 h resolution in March, June, September, and December under low (**top**) and high (**bottom**) solar activity year in the Antarctic.

In order to analyze the diurnal TEC variation in different seasons, the four months March, June, September, and December were selected for representing spring, summer, autumn, and winter in the Arctic, while in the Antarctic representing autumn, winter, spring, and summer, respectively. Figure 14 and Figure 15 show the diurnal change of the C0,0 with the LT in four seasons under low (2010) and high (2014) solar activity conditions, respectively. The TEC was monthly averaged with a 2 h resolution. In the same season, the ionospheric TEC in 2014 was significantly higher than that in 2010 due to the influence of solar radiation, which is consistent with the spatial distributions of TEC shown in Figure 5, Figure 6, Figure 7, Figure 8, Figure 9, Figure 10, Figure 11 and Figure 12. The diurnal variation of the polar ionospheric TEC had lower amplitude than that in the middle and low latitude regions. In the Arctic, the minimum of the ionospheric TEC occurred in winter, and the maximum values appeared in spring and summer under active and calm ionosphere conditions, respectively. In the Antarctic, the ionospheric TEC reached the minimum in winter and maximum in summer. In winter, the variation of the Antarctic ionospheric TEC in one day was less than 5 TECU. In other seasons, the diurnal variations of TEC in the Antarctic were larger than those in the Arctic.

### 4.3. Case Study of Tracking Ionization Patches

At present, the SuperDARN is often used to track ionization patches [30], but the radar network is sparse and cannot completely cover the polar regions in spatial domain. In addition, based on the SCH model, the evolution of ionization patches in the Arctic has been tracked [12]. In this section, a high-precision regional ionospheric model based on GNSS tracking stations was used to track such patches in the Antarctic during a geomagnetic storm, which provided a significant basis for further automatic identification and tracking of these kind of phenomena.

**Figure 16:**
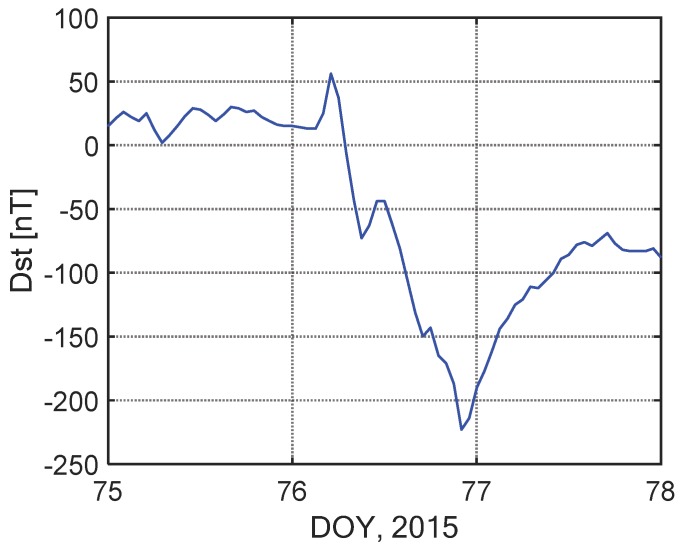
Time series of disturbance storm time (Dst) index on days of year (DOYs) 075–077 in 2015.

The time series of the disturbance storm time (Dst) index is shown in Figure 16. The Dst varied within 0–50 nT during DOY 075, and it dropped down to −225 nT during DOY 076. A geomagnetic storm occurred on DOY 076 (corresponding to Mar 17) in 2015 and it was accompanied by ionization patches.

**Figure 17:**
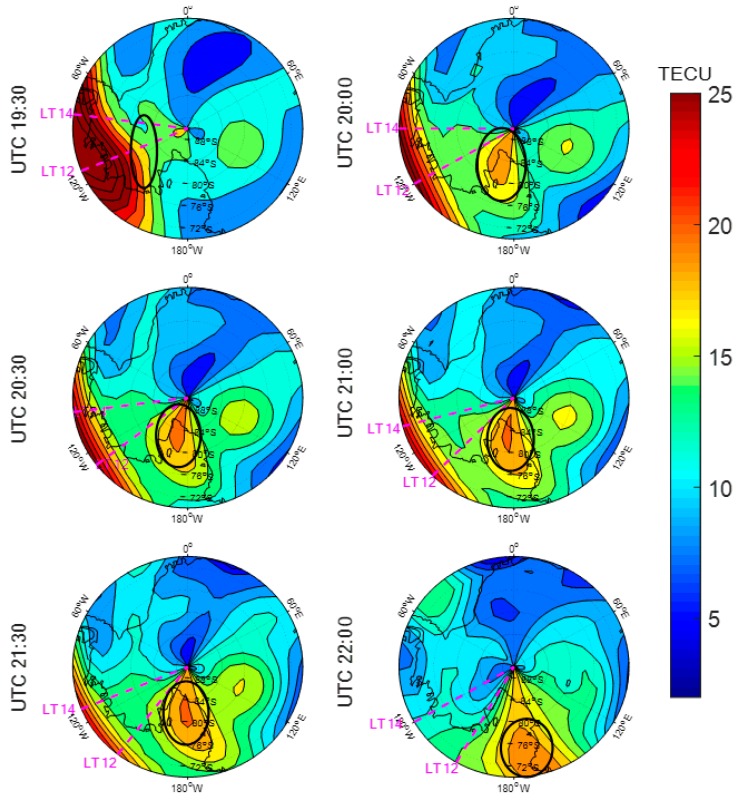
Antarctic ionospheric TEC mapping at UTC (Universal Time Coordinated) from 19:30 to 22:00 on the DOY 076 in 2015. The black ellipses in each subplot highlight the ionization patches.

Figure 17 shows the variation of the Antarctic ionospheric TEC based on the SCH model at UTC from 19:30 to 22:00 during DOY 075 in 2015. The initial condition of the TEC distribution was exhibited at 19:30. At 20:00, a local TEC enhancement (ringed in black) was observed, which appeared in the west close to the geographical South Pole near noon, indicating the formation of an ionization patch. From 20:30 to 21:30, the ionization patch was constantly growing and moving toward low latitude. At 22:00, the ionization patch continued to move toward low latitude. Figure 17 clearly reveals the formation and evolution of an ionization patch in the Antarctic, indicating that the ionospheric model based on the GNSS data is an effective method for monitoring the ionization patches.

## 5. Conclusions

In this paper, four different models were utilized to map the ionospheric TEC in the polar regions based on GPS ground measurements for about 13 years. According to the unique temporal and spatial characteristics of the polar ionosphere, the TEC variation was analyzed considering the disturbed polar ionosphere conditions, including different phases of solar activity, polar days and nights, the WSA, and an ionospheric storm. The variations of the polar TEC during a period of about one solar cycle and the corresponding relationships with the solar activity index of F10.7 were investigated. Moreover, we tracked the evolution of the ionization patch during a geomagnetic storm.

The SCH model had optimal mapping performance for the polar regions compared to other models. During the whole solar cycle, the SCH model had RMS errors of less than 3.0 TECU in the Arctic, while in the Antarctic the RMS errors were between 1.5 and 3.5 TECU. The polar TEC exhibited various changes under different ionosphere conditions. The TEC during the polar day was about twice as much as that during the polar night under similar levels of solar activity. During the polar night, the ionospheric TEC was related to the latitude, and the TEC increased significantly when the latitude became lower in the daytime. In the polar day over the Arctic, the TEC decreased with the decrease of the latitude at night. During the polar day in the Antarctic, the TEC values at UTC from 1:00 to 11:00 did not reach the maximum near the 12:00–14:00 LT time window, and the highest TEC was registered in the Antarctic Peninsula and Weddell Sea because of the influence of the WSA. For one solar cycle, the ionospheric TEC was strongly correlated with the solar activity levels. From 2006 to 2018, the solar activity reached its highest level in 2014, and the polar TEC exhibited the maximum in that particular year. In addition, the ionospheric TEC of the Antarctic had larger amplitude of annual variation than that in the Arctic.

The analysis of the spatiotemporal variations of the polar ionospheric TEC and the study case of ionization patch on DOY 076 in 2015 indicate that the SCH model provides adequate resolution and accuracy to map the polar TEC variation, and it also seems to be very well suited to track some ionization patches; all this could be significant for navigation and positioning in the polar regions.
